# Psychoactive substances and violent offences: A retrospective analysis of presentations to an urban emergency department in Switzerland

**DOI:** 10.1371/journal.pone.0195234

**Published:** 2018-03-29

**Authors:** Evangelia Liakoni, Fabienne Gartwyl, Meret Ricklin, Aristomenis K. Exadaktylos, Stephan Krähenbühl

**Affiliations:** 1 Clinical Pharmacology and Toxicology, Department of General Internal Medicine, Inselspital, Bern University Hospital, University of Bern, Bern, Switzerland; 2 Institute of Pharmacology, University of Bern, Bern, Switzerland; 3 Emergency Department, Inselspital, Bern University Hospital, University of Bern, Bern, Switzerland; 4 Division of Clinical Pharmacology & Toxicology, Basel University Hospital and University of Basel, Basel, Switzerland; “G.d’Annunzio” University, ITALY

## Abstract

**Background:**

Psychoactive substances are often regarded as causal factors contributing to violent injuries, sexual abuse and homicides. While these effects have been demonstrated for some substances (e.g. cocaine), current available data for others are more controversial (e.g. cannabis) or very limited (e.g. ecstasy).

**Aims of the study:**

To collect data on the type and frequency of psychoactive substance use in cases of emergency department (ED) presentations related to interpersonal violence.

**Methods:**

Retrospective study at the University Hospital of Bern, Switzerland, between May 2012 and June 2016. The study covered cases of violent crime associated with psychoactive substances. Cases of isolated ethanol intoxication, suicide attempts, and substance use for medical purposes were excluded.

**Results:**

The study included 103 cases among the 164,846 ED attendances. In the majority of the cases, the type of violence was bodily force (52%) related to urban violence (83%). The mean patient age was 29 years and 79% were male. 63% of the patients reported use of more than one drug; alcohol co-use was reported in 60% of the cases. Besides alcohol, the substances most often reported were cannabis (50%) and cocaine (21%). Alcohol and cannabis was also the most commonly reported substance combination (36% of the total cases). Urine drug screening was performed in 34% of the cases and cannabis and cocaine were the most commonly detected substances (46% and 19%, respectively). There were no cases of novel substances. 23% of the patients were admitted to a hospital ward, 10% to a psychiatric clinic.

**Conclusion:**

Cannabis and cocaine were, besides alcohol, the substances most often reported in ED presentations related to offences of violence. Because of the high prevalence of alcohol co-use, no final conclusions can be drawn on the contribution of single substances.

## Introduction

Psychoactive substances are often regarded as possible contributing causal factors in cases of violent injuries, sexual abuse and homicides. Alcohol, and to a lesser extent illicit drugs, are present in both offenders and victims in many violent events and prevalence of violence is higher among persons who abuse psychoactive substances [1]. Alcohol is one of the most commonly used psychoactive substances in Switzerland [2]. In moderate doses, alcohol induces subjective relaxation and a positive mood, but is often also significantly associated with aggressive behavior and violent crimes [3–5]. Besides alcohol, the recreational use of psychoactive drugs is also common; it is estimated that almost a quarter of the adult population of the European Union have tried illicit drugs at some point in their lives [6]. Moreover, novel psychoactive substances—often with unknown toxicological properties–have been increasingly detected in recent years [7]. Such novel high potency substances, e.g. synthetic cannabinoids and synthetic cathinones (e.g. mephedrone), are more often detected now than in the past, causing new clinical scenarios with aggressive behavior as one of the features of the clinical presentation [8–10].

Data from Switzerland on the acute toxicity of psychoactive substances show that cocaine and cannabis are the psychoactive substances most commonly associated with presentations to the emergency department (ED) [11–13]. Data from both hospitalised and ED patients indicate that cocaine use is related to aggression [4, 14, 15]; this is supported by controlled studies in humans [16] and post mortem toxicological records [17, 18]. The data associating cannabis with violence are more controversial. In some studies frequent use of marijuana was found to be associated with greater likelihood to commit violent offences than no or rare use [19], and the level of aggressiveness has been found to be siginificantly higher in subjects using cannabinoids compared to other substances such as stimulants and sedatives [20]. A positive association between cannabis and interpersonal violence has also been reported in reviews about the consequences of marijuana use based on findings from laboratory, cross-sectional and longitudinal studies [21]. However, there is also data suggesting that acutely intoxicated individuals are less likely to act aggressively, while increased interpersonal aggression may be one of the symptoms of the cannabis withdrawal syndrome [3]. In a cross-sectional study among patients in addiction programs, alcohol and cocaine, but not cannabis, were found to be significantly related to violence [22]. Data on other psychoactive substances (e.g. 3,4-methylenedioxymethamphetamine (MDMA); “ecstasy”) are very limited, so no definitive conclusion is possible. Moreover, for some of those substances, there is an apparent discrepancy between the expected associations and clinical experience. For example, the benzodiazepines would be expected to resemble alcohol, as their pharmacological properties are similar; however, benzodiazepines are often regarded as “antiaggression”drugs [3]. Possible explanations for this discrepancy may include individual differences, such as age, a personal history of mental disorder, and/or dose-related effects.

There are currently only limited data on ED presentations related to violent behavior associated with the use of psychoactive substances other than alcohol. Data from Switzerland are available only from a case-crossover analysis from the University Hospital of Lausanne, in which the potential impact of alcohol and cannabis use on the risk of injury (in general and not only in relationship to violent assaults) was studied by using a questionnaire [23]. At the moment, there are no data available on self-reported and analytically confirmed substance use in patients with violent behavior and assaults presenting in urban EDs in Switzerland. Since this is a frequent problem, the present study aimed to describe the presentations related to violent assaults in association with psychoactive substance use at a large urban ED in Bern, Switzerland, over a period of four years and two months. Our main objective was to collect data on the type and frequency of psychoactive substance use in cases of ED presentations related to interpersonal violence.

## Materials and methods

This retrospective study was approved by the local ethics committee (No. 2016–01394). The study covered all patients admitted to the ED at Bern University Hospital between May 2012 and June 2016 because of violent offences associated with psychoactive substance use. The ED of the Inselspital, Bern University Hospital, serves a catchment area of about 2 million people in the Canton of Bern, with about 40,000 emergency admissions a year (≥16 years of age) and is both a primary care facility (walk-in patients) and a tertiary referral centre for other hospitals in the area.

Cases were retrieved from the electronic patient chart database using a comprehensive full-text search algorithm. In brief, the automatic search identified all cases mentioning violence, violent, assault, violent conduct, violent offence, fight, attack, choke, strangle, beat, stab, physical abuse, or related terms, including abbreviations and misspellings. The charts for all cases were reviewed; only cases were included that presented because of symptoms associated with violent offences (offenders or victims) and concomitant use of psychoactive substances (recreational drugs alone or in combination with alcohol). A violence offence was defined as physical pain or damage intentionally inflicted by another person. A recreational drug was defined as a psychoactive compound that was taken for the purpose of recreational activities rather than for medical or work purposes or for self-harm. The recreational drug(s) associated with the presentation were identified on the basis of at least one of the following findings: the patient’s self-report, information retrieved from witnesses, analytical results. Data extraction was performed by one of the authors for the entire study. The study excluded cases of isolated ethanol intoxication, cases in which the patient left the ED before being seen by the ED staff, cases with substance use as suicide attempt, cases with substance use for medical purposes (e.g. substitution therapy with opioids) unless the substances were used for recreational purposes differently than prescribed (e.g. route of administration i.v. instead of p.o., higher dose), and cases attending the ED for a follow-up and not in the context of an acute intoxication. In accordance with our study question, which was the investigation of possible associations between interpersonal violence and recreational drug use, cases of self-harm and self-aggression were not included, as the underlying factors (e.g. mental disorders, terminal illness) and the dose used can greatly differ from the cases included in the current study. Cases of substance withdrawal were also not included, because violent behavior in such cases can result from avoiding withdrawal effects (e.g. desperate drug-seeking) and not from the pharmacological properties of the substance itself [1].

The patient demographics (age, sex, hour and week day of ED admission), the psychoactive substances involved as reported by the patient or witnesses, the route of hospital admission (e.g. ambulance, police), the clinical effects, and clinical outcome were recorded. A urine drug screening test using an immunoassay (Triage® TOX Drug Screen, Alere, Cologne, Germany) was used to screen for amphetamines, barbiturates, benzodiazepines, cocaine, methadone, methamphetamines (including MDMA), opiates, phencyclidine (PCP), tricyclic antidepressants, and tetrahydrocannabinol (cannabis). The cut-off level was 1000 ng/mL for amphetamines, methamphetamines, and tricyclic antidepressants, 300 ng/mL for barbiturates, benzodiazepines, cocaine, methadone, and opiates, 50 ng/mL for cannabis, and 25 ng/mL for PCP [24]. Ethanol blood levels were estimated from the osmolar gap using the following equations: serum ethanol (g/L) = (serum osmolality–(2 * sodium (mEq/L) + glucose (mmol/L) + blood urea nitrogen (mmol/L)) / (1.25 * 21.71) and ethanol concentration (g/kg) = serum ethanol (g/L) / (1.236) ‰ [25, 26].

## Results

During the study period, from May 2012 to June 2016, there were 164,846 ED presentations. Of these, 1,945 cases were associated with violent offences (1,697 with the patient as victim and 248 with the patient as offender); among these, alcohol alone was involved in 386 cases and psychoactive substances alone or in combination with alcohol (i.e. included cases) in 103 cases.

The patient characteristics of the 103 included cases are shown in Table 1.

**Table 1:** Patient and case characteristics.

	Number of cases, N = 103 (%)
Male	81 (79)
Female	22 (21)
**Age (years)**	
≤20	15 (15)
21–30	52 (50)
31–40	25 (24)
41–50	8 (8)
>50	3 (3)
**Area of origin**	
Switzerland	72 (70)
Europe other than Switzerland	10 (10)
Africa	13 (13)
Asia	6 (6)
South America	2 (2)
**Medical history**	
Chronic substance abuse	37 (36)
Known psychiatric disorder	20 (19)
**Time of presentation**	
Night arrival (20:00–8:00 h)	77 (75)
Weekend arrival (Friday 17:00 h–Monday 8:00 h)	70 (68)
**Substance use**	
Reported ethanol co-ingestion	62 (60)
Reported use 1 drug	23 (22)
Reported use >1 drug	65 (63)
No information available (e.g. coma, uncooperative)	15 (15)
Urine drug screening test performed	35 (34)
**Type of offence**	
Urban violence	86 (83)
Domestic violence	14 (14)
Rape	3 (3)

**Fig 1:**
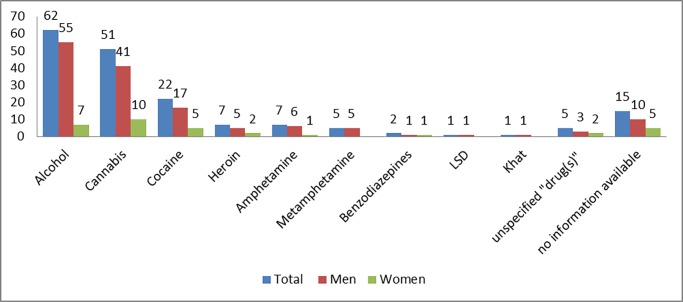
Reported substance use (count of cases).

The mean patient age was 29 years (range 16–64); 34% were brought to the ED by ambulance and 17% under police escort. The ED admission was associated with inflicted injuries in 16 cases (16%). In 94 cases (91%), the patient suffered an injury (both suffered and inflicted injuries in some cases). Schizophrenia was documented in 7 of the patients with a known psychiatric disorder. Among the 65 patients who reported use of more than one drug, 56 reported use of two drugs, while use of three and five drugs was reported in 8 and 1 cases, respectively. In 88 cases, the information was based on the patient’s self-reported use, in 6 cases the patient provided information on the substance use of the other person involved in the offence (additionally to their own substance use in some cases), and in 13 cases the information was derived from the urine drug screening test. Fig 1 shows the reported substances involved.

**Fig 2:**
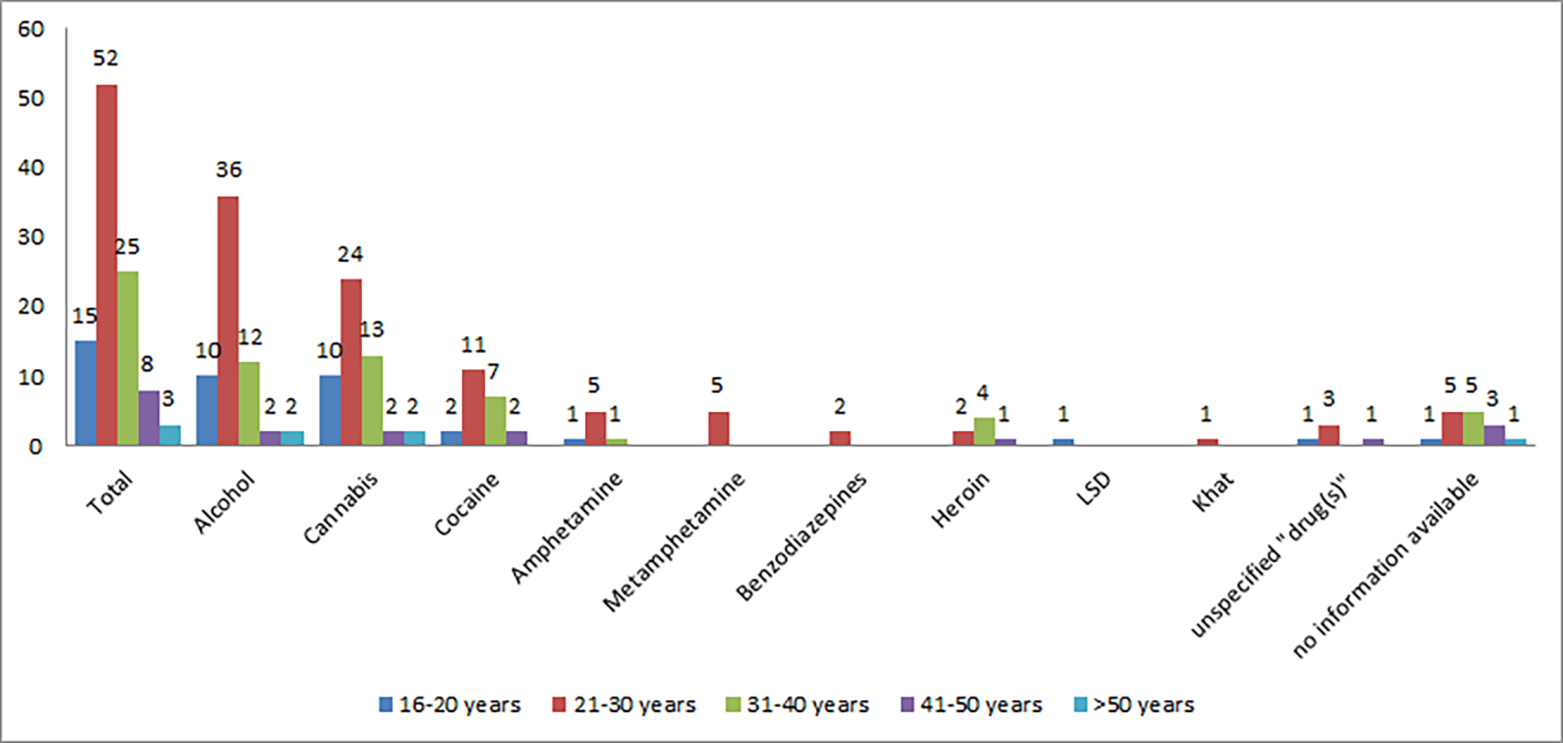
Age groups and reported substance use (count of cases).

The reported substances in the different age groups are shown in Fig 2.

**Fig 3:**
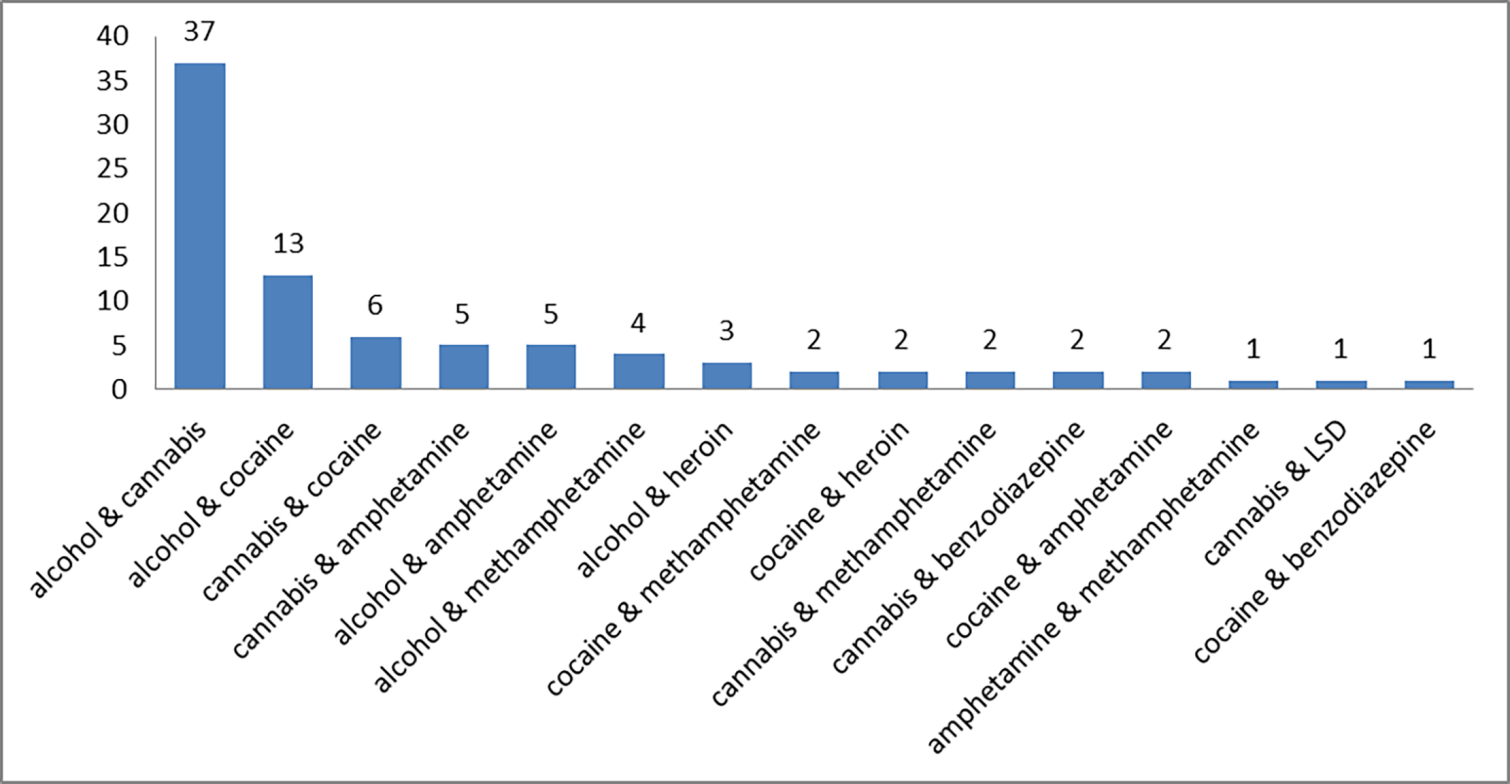
Frequency of reported substance combinations.

Table 2 and Fig 3 show the reported substances and the frequency of their combination in cases with reported use of more than one substance.

**Table 2:** Reported substances in cases with use of more than one substance.

	alcohol	cocaine	cannabis	amphetamine	methamphetamine	benzodiazepines	heroin	LSD
**plus alcohol**	x	13	37	5	4	0	3	0
**plus cocaine**	13	x	6	2	2	1	2	0
**plus cannabis**	37	6	x	5	2	2	0	1
**plus amphetamine**	5	2	5	x	1	0	0	0
**plus metamphetamine**	4	2	2	1	x	0	0	0
**plus benzodiazepines**	0	1	2	0	0	x	0	0
**plus heroin**	3	2	0	0	0	0	x	0
**plus LSD**	0	0	1	0	0	0	0	x

**Fig 4:**
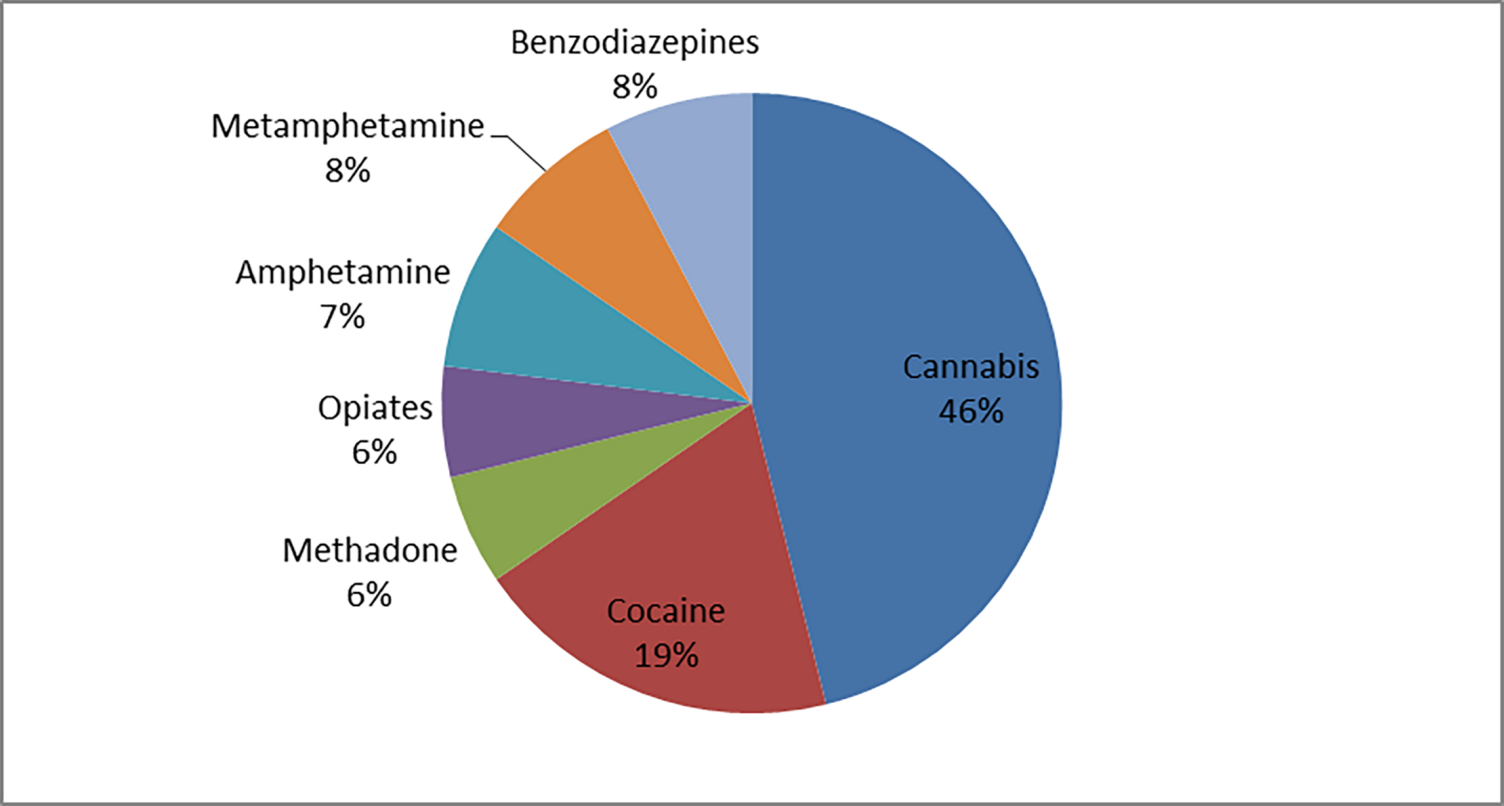
Substances detected with the urine drug screening immunoassay (n = 35).

A urine drug screening test was performed in 34% of the cases (Table 1), of these, the analytical results were identical with the patient’s report in 6 of the 35 cases (17%). The detected substances are shown in Fig 4.

**Fig 5:**
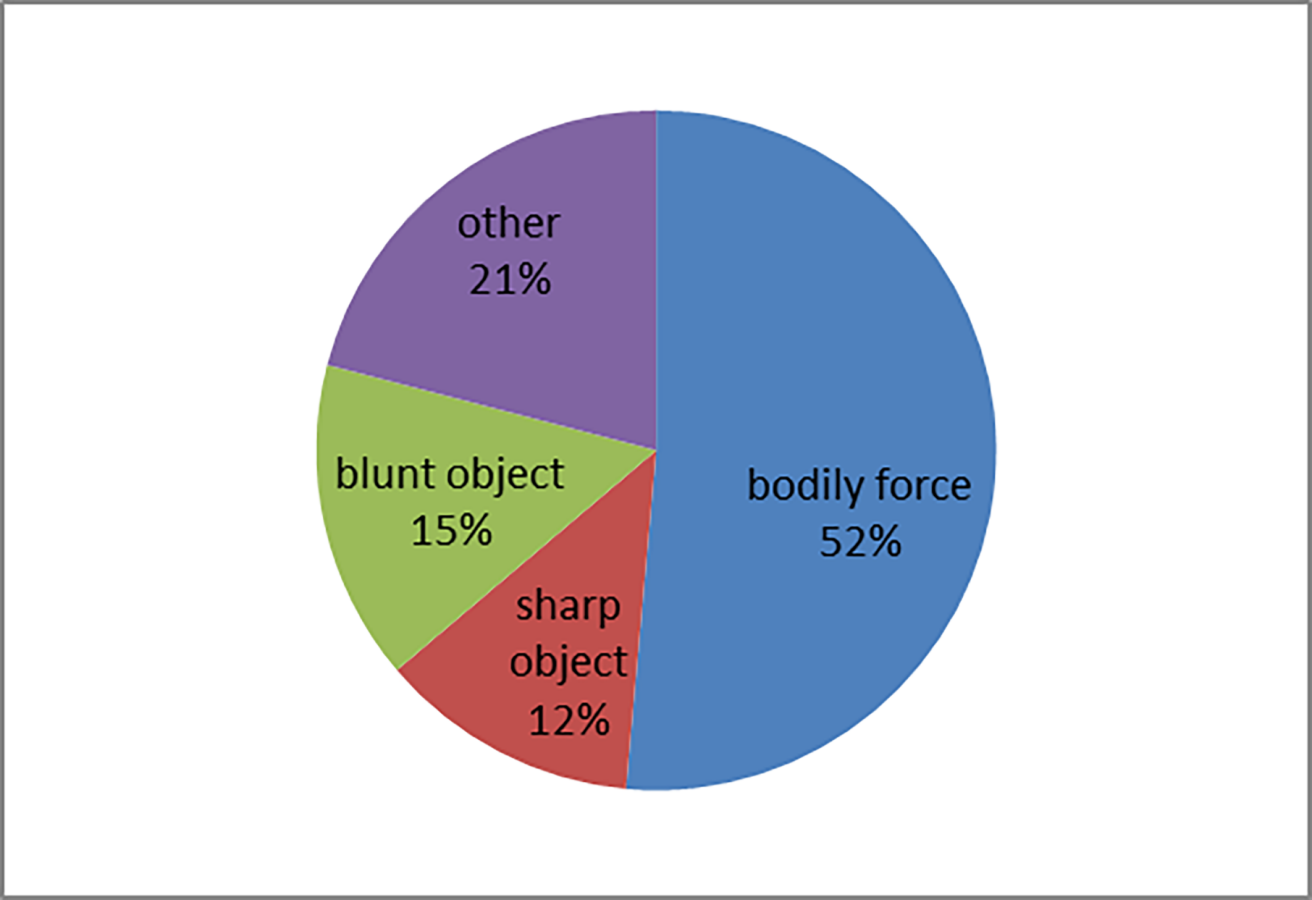
Type of violence used.

In 8 cases (8%), participation in a methadone substitution program was documented. Alcohol co-use was confirmed analytically in 23 (22%) cases; the median serum alcohol concentration was 1.53 ‰ (range 0.09–4.6). The type of violence used is shown in Fig 5.

**Fig 6:**
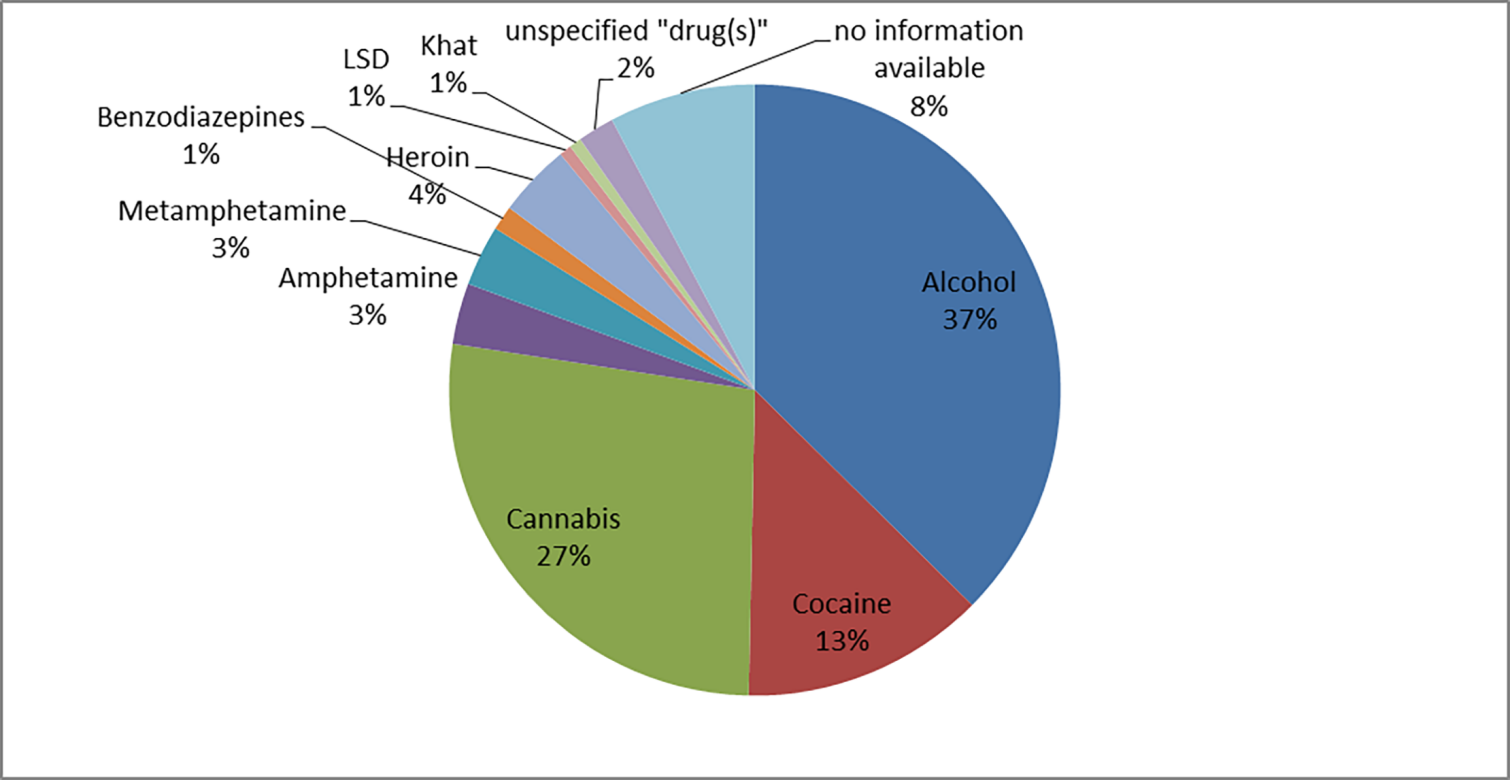
Reported substance use in cases of urban violence (n = 86).

**Fig 7:**
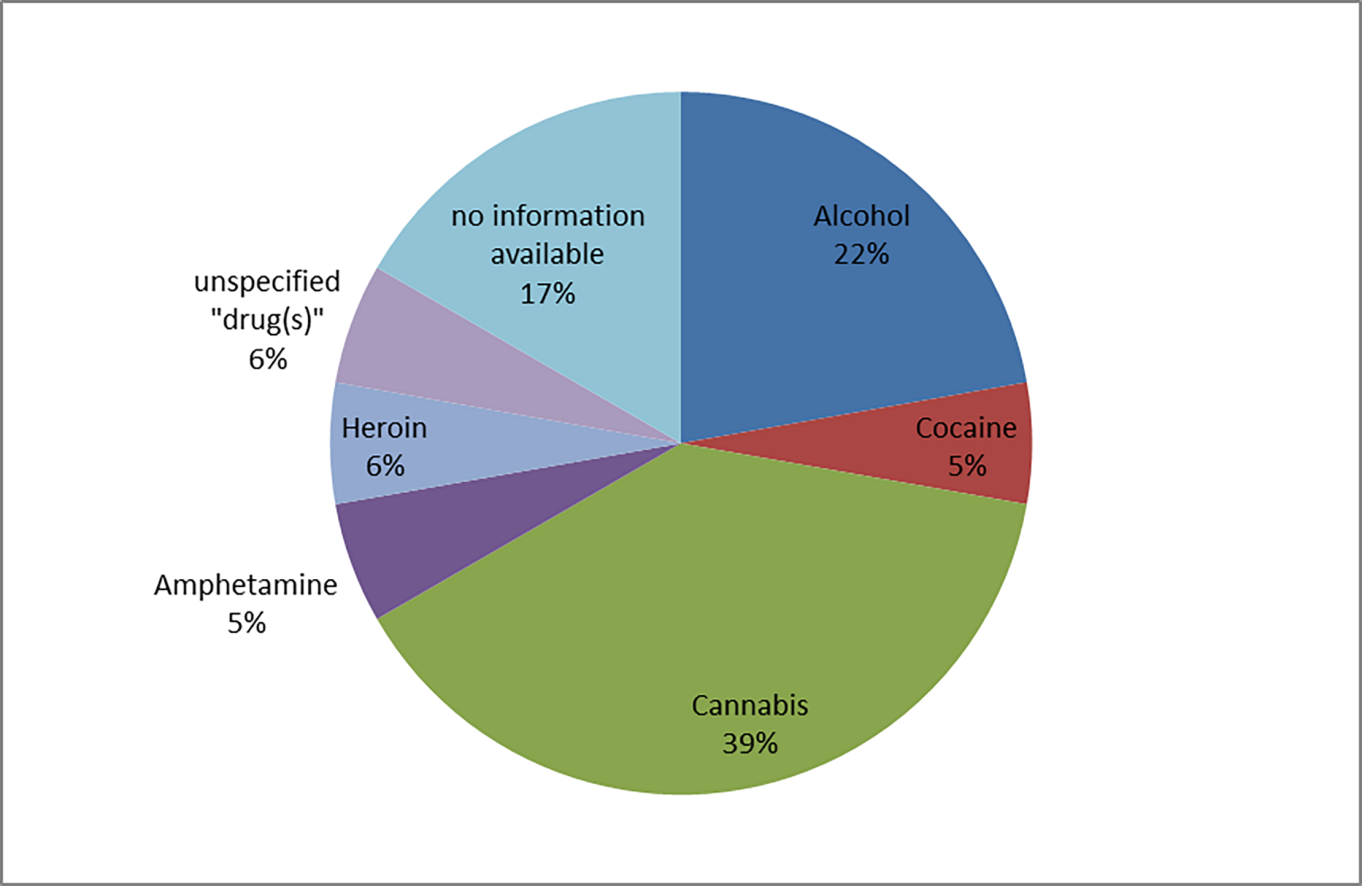
Reported substance use in cases of domestic violence (n = 14).

Figs 6 and 7 show the frequency of the substances involved in cases of urban and domestic violence, respectively. In the three rape cases, the reported substances were cannabis, cocaine, and amphetamine.

There were no admissions to the intensive care unit; 24 patients (23%) were admitted to a hospital ward, 10 patients (10%) to a psychiatric clinic. Substances involved in cases in which the patient was admitted to a hospital ward included alcohol (15/24 cases), cannabis (10/24), cocaine (3/24), methamphetamine (2/24), amphetamine, benzodiazepine, heroin and khat (each 1/24).

## Discussion

The present study describes the type and frequency of psychoactive substance use in cases of ED presentations related to interpersonal violence. Besides alcohol, the substances most often involved were cannabis and cocaine, while other psychoactive substances were comparatively infrequently reported and/or detected. In most cases, the patient was male, between 21 and 30 years old, and was brought to the ED during the night in a weekend after an urban violence offence. In more than one third of the cases, the personal history of the patient included chronic substance abuse, and approximately 1/5 of the patients suffered from a psychiatric disorder. In most cases, use of more than one substance (mostly 2) was reported; the most commonly reported combination was alcohol and cannabis. Urine drug screening was relatively rarely performed (1/3 of the cases). Most admissions to a hospital ward were related to alcohol use, followed by cannabis and cocaine.

In our findings, cannabis seems to play a dominant role after alcohol for presentations related to violent offences. However, these estimations should be interpreted with caution, as cannabis is the most commonly used drug in Europe [27] and the prevalence of use must also be considered. Furthermore, as cannabis was also the most commonly detected substance in our study, it must be stressed that the urine test can remain positive for days or weeks after cannabis use [28]. Thus, a positive result does not necessarily indicate cannabis use in the hours prior to presentation. In another study at the same ED investigating cases with acute recreational drug toxicity [13], most presentations were related to cocaine (29%) and cannabis (26%). In our study, the self-reported use of cannabis was related to violent offences in half of the cases, but cocaine in only 21%. Thus, although cannabis and cocaine seem to lead more or less equally often to ED presentations linked to their acute drug toxicity (e.g. sympathomimetic and psychiatric disorders), there seems to be more frequent involvement of cannabis in cases related to interpersonal violence. However, cannabis was combined with alcohol in the great majority of the cases (37 out of the 51) in our study, which was also the most commonly reported combination of substances. It is therefore not clear if cannabis, alcohol, or their combination was a contributing factor in these cases. Synergy between alcohol and other drugs has been shown in studies focusing on intentional injuries [29], while other studies found that co-use of other drugs did not enhance the association between alcohol and violent injuries by the patient [30]. The quantity of alcohol ingested and the frequency of alcohol consumption are also of importance in such cases. Although this information is usually not known, new emerging trends such as binge drinking and drunkorexia should be taken into account for future studies, as they have been found to be associated with the concomitant use of other drugs such as cocaine and novel substances [31]. Moreover, it is likely that the amount and the type of cannabis used also played a role in the development of aggressive behavior in some patients. The concentration of tetrahydrocannabinol (THC), the primary psychoactive component of cannabis that has been shown to trigger paranoia in vulnerable individuals [32], may be important regarding the development of aggressions. It has been proposed that low THC doses may increase, while moderate and high doses may suppress aggressive behavior [3]. In order to further investigate these aspects, we would require a much larger number of monointoxications and with more information available. However, this is very difficult to achieve in a real life ED setting, where more than one drug and also unknown substances are used by a substantial number of patients [11–13, 33]. Using data collected in 14 European centres, the European Drug Emergencies (Euro-DEN) project [34] aimed to describe the characteristics of cannabis monointoxication on the basis of 36 lone-cannabis cases among 356 cannabis intoxications [35]. In this small number of patients, agitation and/or aggression was reported in 8 cases (22%) and sedation was the treatment most commonly received (in 6 cases, 17%). However, because agitation and/or aggression were reported as one clinical feature, it is not possible to distinguish in how many cases aggressive behavior was actually present. In the study from Lausanne, which investigated the potential impact of alcohol and cannabis use on the risk for injury [23], intentional injuries were reported in 9% of the 486 total cases. However, it is not known how many patients used only cannabis and the authors admit that their findings were limited by the small sample size of the cannabis users.

An interesting finding in our study was the absence of reported and/or detected novel psychoactive substances, despite previous findings suggesting an increased risk of violence associated with abuse of such substances [36]. Novel psychoactive substances, also known as “designer drugs” and “legal highs”, are usually analogues or derivatives of controlled substances, produced in order to circumvent regulations and their use has rapidly increased in recent years [7]. Novel substances were also infrequently reported in several recent studies on similar patient populations [11–13]. It is therefore possible that those substances were not found in our study because of their infrequent use in Switzerland. Other possible reasons could be that the toxicological properties of the novel substances are not associated with aggressive behavior and/or that they were involved in some cases but not reported. The immunoassay drug tests routinely used at EDs are rapid and easy to use, but provide only preliminary information about the substance(s) used. These immunoassays have many limitations, including the fact that they typically cannot detect novel substances. Most novel substances could be detected with other analytical methods (e.g. liquid or gas chromatography combined with mass spectrometry), but costs, run time, and need for specialized personnel limit their use in the ED setting. Therefore, if none of these additional analytical methods is used and the novel substance is not self-reported, its use will remain undetected.

A urine drug screening test was performed in the minority of the cases in our study (approximately in one third of the patients), and in less than 20% of those tests the results matched the patient’s report. Possible reasons for the poor agreement between reported and detected substances may be that some substances ingested as regular medication or administered in the ED (e.g. benzodiazepines) were overrepresented in the analytical results. Similarly, substances with a long half-life that were ingested a long time before the acute episode (e.g. cannabis) could also have been overrepresented in the urine samples. On the other hand, substances which can be detected only during a very short period of time (e.g. gamma-hydroxybutyrate (GHB)) or cannot be detected with the immunoassay test used in the study (e.g. LSD, novel substances) may have gone undetected. A further important limitation of the drug urine immunoassay is that it can yield false-positive (e.g. cross reactivity with other compounds) or false-negative results (e.g. concentrations below the cut-off). These limitations should be considered when interpreting analytical results and they are probably one of the reasons for the low number of drug screening tests performed in our study, as management of cases presenting at the ED is mostly based on the self-reported substance use and the clinical presentation. Further possible reasons for the low number of drug screening tests are that they might not have been indicated in some cases (e.g. if the self-report matched the clinical presentation and no changes regarding further management based on the analytical confirmation were expected), or that the collection of a urine sample was refused by the patient or was not possible due to logistic reasons (e.g. need for a quick admission to a psychiatric clinic).

On the basis of the patient characteristics seen in our study, a profile can be drawn of the typical patient presenting at the ED because of interpersonal violence and possible association with psychoactive substance use. The young age of the majority of the patients as well as the time of presentation (night time and/or weekend) suggests use in a recreational setting at off-peak hours. Most patients were men, which could indicate either that men are more frequently involved in violent offences and/or that they consume psychoactive substances more often than women. Interestingly, some studies have reported sex specific associations, with a greater risk of violence after cocaine use for female and after heroin use for male patients [37]. Most cases in our study involved urban violence, with alcohol as the substance most commonly reported, followed by cannabis and cocaine. In cases of domestic violence, cannabis was most commonly reported, followed by alcohol and heroin. It is possible that the environment in which drug use usually takes place contributes to those findings (e.g. cocaine use probably more common in bars/nightclubs than at home). However, because of the small number of cases with domestic violence (n = 14) and the frequent combination of substances, no final conclusion can be drawn on the different substances involved in urban and domestic violence.

Associations between violent behavior and psychoactive substance use may not only be linked to the substances’ toxicological properties, but also to their illegal status, as users are often forced to come in contact with an uncontrolled and potentially aggressive environment. Thus, violent crimes can be committed to gain access to illicit substances or to resolve conflicts and may not be associated with the drug’s direct pharmacological effects (intoxication), neurotoxicity (caused by prolonged use) or withdrawal effects [3]. Moreover, a vicious cycle relationship has been described in which substance use increases the risk of future assault and assault increases the risk of subsequent substance use [38]. Such factors should also be considered when investigating possible reasons for the relationship between psychoactive substance use and aggression. Interestingly, sole use of a legally available psychoactive substance—alcohol—was involved in presentations associated with violent offences in almost 4 times more cases in our study than psychoactive substances alone or in combination with alcohol (386 vs. 103 cases). This could be associated with the specific psychoactive properties of alcohol or with its widespread use. It is therefore unclear whether future legalization of other psychoactive substances would lead to fewer violent offences (because of the elimination of the unregulated environment) or to more offences due to higher consumption. It is also possible that sociodemographic characteristics, psychiatric disorders or other factors may make some individuals more likely to use a specific substance. This could then be the cause of changes in the prevalence of violence rather than the inherent properties of the substances [39].

One limitation of our study was that the patients were in some cases the victim of the offence and that there was no information on the substance use by the perpetrator. However, in cases of violent offences it is not always easy to differentiate between victim and perpetrator, as it is possible that the offence was initiated by the person who suffered the injuries. Moreover, use of psychoactive substances appears to be associated with violent offences and crimes even if the user is not the perpetrator; according to studies on the epidemiology of rape among women using crack cocaine, more than 80% reported that they were high on crack when the crime occurred, more than 60% had suffered a physical attack since initiating crack use [40], and women’s use of illicit drugs has been shown to be associated with violence on the part of other persons [15]. Further limitations of our study include the limitations of the drug screening immunoassay mentioned above as well as the low number of cases in which the immunoassay was performed. Although a medical history (including noxae) is obtained regularly from all patients presenting at the ED, with additional drug screening if medically indicated, it cannot be excluded with certainty that this procedure was not followed completely in some of the cases during the study period (e.g. uncooperative patients). Furthermore, there were some missing data (the initial patient data were not recorded in a standardised manner), individual characteristics, psychiatric disorders or substance withdrawal may have contributed to the expression of violence in some cases, alcohol co-use was reported in the majority of the cases, and data from only one ED may reflect local trends and may not be representative. Due to the descriptive nature of the study, possible implications are investigated hypotheses and not causal links. Despite these limitations, our study is, to our knowledge, the first one to investigate the possible association of different psychoactive substances, including novel substances that have been increasingly detected in recent years, with violent behavior and assaults using both self-reports and analytical results from a large urban ED in Switzerland over a time period of more than four years. At the same time, our findings provide information about the frequency of presentations, type of violence, combination of substances and their distribution in age groups.

In conclusion, besides alcohol, cannabis and cocaine were the substances most often reported in ED presentations related to violent offences in our study. However, alcohol co-use was reported in the majority of the cases and alcohol-cannabis was the most commonly reported substance combination. Therefore, it is not clear to what extent each of the psychoactive substances alone could have contributed to the expression of interpersonal violence. Future multicenter studies with a prospective study design and focus on monointoxications would help to eliminate bias such as individual characteristics and to understand and evaluate the contribution of each psychoactive substance separately. However, combinations are more common and this is therefore very difficult to accomplish in a real life ED setting.
